# Metabolic engineering of *Rhodotorula toruloides* for resveratrol production

**DOI:** 10.1186/s12934-022-02006-w

**Published:** 2022-12-24

**Authors:** Mengyao Zhang, Qidou Gao, Yijuan Liu, Zhumei Fang, Zhiwei Gong, Zongbao K. Zhao, Xiaobing Yang

**Affiliations:** 1 College of Enology, Northwest A&F University, Yangling, Xianyang, 712100 Shaanxi China; 2 Division of Biotechnology, Dalian Institute of Chemical Physics, Chinese Academy of Sciences, 457 Zhongshan Road, Dalian, 116023 China; 3 School of Chemistry and Chemical Engineering, Wuhan University of Science and Technology, 947 Heping Road, Wuhan, 430081 China

**Keywords:** *Rhodotorula toruloides*, Resveratrol, Metabolic engineering, Cell factory

## Abstract

**Background:**

Resveratrol is a plant-derived phenylpropanoid with diverse biological activities and pharmacological applications. Plant-based extraction could not satisfy ever-increasing market demand, while chemical synthesis is impeded by the existence of toxic impurities. Microbial production of resveratrol offers a promising alternative to plant- and chemical-based processes. The non-conventional oleaginous yeast *Rhodotorula toruloides* is a potential workhorse for the production of resveratrol that endowed with an efficient and intrinsic bifunctional phenylalanine/tyrosine ammonia-lyase (*Rt*PAL) and malonyl-CoA pool, which may facilitate the resveratrol synthesis when properly rewired.

**Results:**

Resveratrol showed substantial stability and would not affect the *R. toruloides* growth during the yeast cultivation in flasks. The heterologus resveratrol biosynthesis pathway was established by introducing the 4-coumaroyl-CoA ligase (*At*4CL), and the stilbene synthase (*Vl*STS) from *Arabidopsis thaliana* and *Vitis labrusca*, respectively. Next, The resveratrol production was increased by 634% through employing the cinnamate-4-hydroxylase from *A. thaliana* (*At*C4H), the fused protein *At*4CL::*Vl*STS, the cytochrome P450 reductase 2 from *A. thaliana* (*At*ATR2) and the endogenous cytochrome B5 of *R. toruloides* (*Rt*CYB5). Then, the related endogenous pathways were optimized to affect a further 60% increase. Finally, the engineered strain produced a maximum titer of 125.2 mg/L resveratrol in YPD medium.

**Conclusion:**

The non-conventional oleaginous yeast *R. toruloides* was engineered for the first time to produce resveratrol. Protein fusion, co-factor channeling, and *ARO4* and *ARO7* overexpression were efficient for improving resveratrol production. The results demonstrated the potential of *R. toruloides* for resveratrol and other phenylpropanoids production.

**Supplementary Information:**

The online version contains supplementary material available at 10.1186/s12934-022-02006-w.

## Introduction

Resveratrol possesses excellent biological activities and pharmacological properties, which has extensive applications in the chemical, pharmaceutical, food, and cosmetic industries [1, 2]. However, the plant-based extraction could not satisfy ever-increasing market demand, while the chemical synthesis is impeded by the existence of toxic impurities, generated during multiple-step complex reactions, for industrial scale applications [3, 4]. Microbial cell factory offers an alternative approach for resveratrol production since it has advantages like eco-compatibility, and high stereo-selectivity [5].

Microbial production of resveratrol could be achieved through the shikimate and aromatic amino acid (AAA) pathway via recruiting cinnamate-4-hydroxylase (C4H), 4-coumaroyl-CoA ligase (4CL), and stilbene synthase (STS) with L-phenylalanine (L-Phe) as the direct precursor or introducing 4CL and STS with L-tyrosine (L-Tyr) as the starter. To date, microbes like *Escherichia coli*, *Yarrowia lipolytica* and *Saccharomyces cerevisiae* have been intensively explored for resveratrol production [5]. For *ex novo* production, the best recombinant *E. coli* produced 2.3 g/L resveratrol from *p*-coumaric acid [6] while for de novo biosynthesis, 0.8 g/L and 22.5 g/L resveratrol were obtained with *S. cerevisiae* and *Y. lipolytica* in bench-scale production, respectively [7, 8].

The non-conventional oleaginous yeast *R. toruloides* is attractive for producing various value-added chemicals including oleochemicals, terpenoids and sugar alcohols from low-cost feedstock [9–11]. *R. toruloides* might be also a potential workhorse for aromatic compounds that it is endowed with an efficient and intrinsic bifunctional *Rt*PAL. As an oleaginous yeast, it should also provide substantial malonyl-CoA and erythrose-4-phosphate (E4P) for aromatic compounds biosynthesis since they are highly required for the fatty acid biosynthesis and NADPH generation during lipid accumulation. Importantly, the *Rt*PAL has been demonstrated efficient in catalyzing L-Phe to *trans*-cinnamic acid (*t*-CA), and L-Tyr to *p*-coumaric acid (*p*-CA) to support the resveratrol production [12]. To date, no attempts have been made to produce phenylpropanoid compounds, such as resveratrol, in *R. toruloides*.

**Fig. 1:**
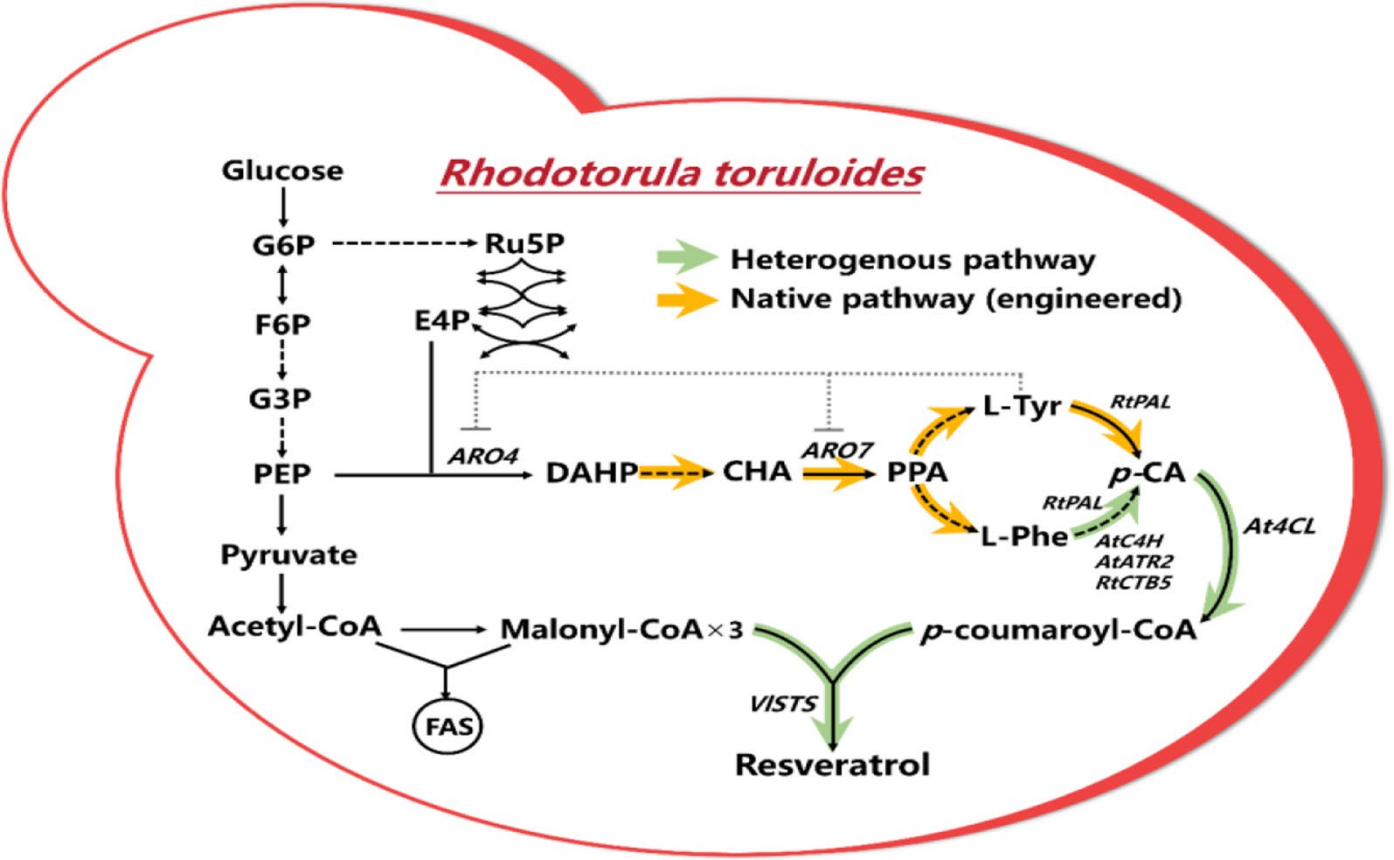
Engineering *Rhodotorula toruloides* to produce resveratrol. The heterogeneous pathway is green, and the engineered native pathway is yellow. The grey dashed line indicated feedback inhibition. G6P, glucose-6-phosphate; F6P, fructose-6-phosphate; G3P, glyceraldehyde-3-phosphate; PEP, phosphoenolpyruvate; Ru5P, ribulose-5-phosphate; E4P, erythrose-4-phosphate; DAHP, 3-deoxy-arabino-heptulonate-7-phosphate; CHA, chorismic acid; PPA, prephenate; L-Phe, L-phenylalanine; L-Tyr, L-tyrosine; p-CA, p-coumaric acid; FAS, fatty acid synthase; ARO4, 3-deoxy-7-phosphoheptulonate synthase; ARO7, chorismate mutase; PAL, L-phenylalanine ammonia-lyase; *At*C4H, cinnamic acid hydroxylase from *A. thaliana*; *At*4CL, 4-coumarate-CoA ligase from *A. thaliana*; *Vl*STS, stilbene synthase from *Vitis labrusca*; *At*ATR2, cytochrome P450 reductase from *A. thaliana*; *Rt*CYB5, cytochrome B5 from *R. toruloides*

To tap its potential for aromatic compound production, the oleaginous yeast *R. toruloides* was engineered to produce resveratrol as an example by introducing *At*4CL and *Vl*STS (Fig. 1). Subsequently, the production was significantly increased via critical genes overexpression, protein fusion, and cofactor channeling. Finally, the maximum titer was improved to 125.2 mg/L. The present study demonstrated that *R. toruloides* could be explored as a platform for phenylpropanoid bioproduction.

## Materials and methods

### Strains, media and cultivation

*E. coli* DH5α was used for plasmid construction and propagation. *Agrobacterium tumefaciens* AGL1 was used for *R. toruloides* transformation. The *R. toruloides* NP11 is the haploid of *R. toruloides* CGMCC 2.1389 that was isolated in Prof. Zongbao Zhao’s Lab. *E. coli* and *A. tumefaciens* cells were cultivated in Luria Bertani (LB) medium (10 g/L tryptone, 5 g/L yeast extract, 10 g/L NaCl, and supplemented with 50 μg/mL kanamycin), and kept at 200 rpm, 37–30 °C, respectively. *R. toruloides* was cultivated at 28 °C, 180 rpm in the YPD medium (20 g/L glucose, 20 g/L peptone, and 10 g/L yeast extract). The selection YPD medium used was supplemented with 50 μg/mL of Nourseothricin (Ntc) or Hygromycin B (Hyg) as needed. For the preparation of solid medium, 2% (w/v) agar was added into liquid LB and YPD. Induction medium (IM) containing 200 μL acetosyringone was prepared as described [13]. Briefly, the IM medium contained 10 mmol/L K_2_HPO_4_, 10 mmol/L KH_2_PO_4_, 2.5 mmol/L NaCl, 2 mmol/L MgSO_4_, 0.7 mmol/L CaCl_2_, 9 μmol/L FeSO_4_, 4 mmol/L (NH_4_)_2_SO_4_, 10 mmol/L Glucose, pH 7.0.

### Plasmid construction

The heterologous genes were codon-optimized according to the *R. toruloides* preference and synthesized by Synbio Technologies (Suzhou, P. R. China). All vectors used in this study were derived from the binary vector pZPK [13]. The DNA ligation kit (Takara) and In-Fusion HD cloning kit (Takara) were employed for plasmid) construction, following its user instruction. The PCR-based mutation was used for obtaining protein mutants. All the vectors and primers used in this study were summarized in Additional file 1: Tables S1 and S2, respectively.

### Transformation and verification

*Agrobacterium*-mediated transformation (ATMT) was modified according to the protocol reported by Lin et al. [13]. Briefly, the correct binary vector was transformed into *A. tumefaciens* AGL1 cells by electroporation, and selected on LB agar plates containing 50 μg/mL kanamycin. The *A. tumefaciens* cells carrying the binary vectors and the *R. toruloides* cells were cultivated at 28 °C until OD_600_ reached 2. Both cells were washed twice, and diluted to OD_600_ = 0.4–0.6 with distilled water. The cell suspensions were mixed with a ratio of 1:1 (v/v). Then, 200 μL of the mixture was spread onto the filter paper placed on the IM plate and incubated at 25 °C for 36 h. Subsequently, the filter paper was transferred onto the selection YPD plate for screening transformants harboring the Ntc or Hyg resistance markers (supplemented with cefotaxime and corresponding antibiotics (Nourseothricin or Hygromycin B) and incubated at 30 °C until colonies appeared. The transformants were randomly selected and streaked onto selecting plates for five generations to certify their stability.

### Cultivation in shake flask

The *R. toruloides* was seeded into 50 mL test tubes containing 5 mL YPD liquid medium supplemented with 50 μg/mL antibiotics if needed, and cultivated under 28 °C, 180 rpm for 48 h. Then, the seed cultures were inoculated into 50 mL medium with the initial OD_600_ = 0.5 in 250 mL Erlenmeyer flasks and grown at 28 °C, 180 rpm for 96 h. Unless otherwise stated, the fermentation in 250 mL Erlenmeyer flasks were loaded with 50 mL YPD medium. To test its stability during yeast fermentation, 0.5 mM resveratrol was added to replace the glucose in the YPD medium.

### Analytical methods

The cell density was tested with UV–Vis spectrophotometer EVOLUTION 220 (Thermo Fisher Scientific, USA). The d-glucose was quantified by the SBA-40C biosensor (Shandong Province Academy of Sciences, Jinan, China). The resveratrol production capacity of the transformants was analyzed in terms of maximum and averaged titers, since the ATMT strategy leads to random integration in the genome [14]. The analysis of resveratrol and *p*-coumaric acid was performed as described by Wang et al. [16]. 3 mL of fermentation samples were mixed with 3 mL of ethyl acetate, vortexed thoroughly, and centrifuged at 12,000 rpm, for 5 min at 4 °C. The supernatant was dried with RapidVap (Labconco, USA) at room temperature, re-dissolved in 300 μL acetonitrile, and filtrated by a 0.22 μm membrane before high-performance liquid chromatography (HPLC) analysis. The Shimadzu LC-2030 PLUS HPLC system is equipped with a Waters T-nature C18 column (4.6 × 250 mm, 5 μm) at 306 nm under isocratic elution of 65% (1% acetic acid aqueous) and 35% (acetonitrile) over 4.3 min (p-coumaric acid), 5.9 min (resveratrol). The column working temperature was kept at 35 °C, and the injection volume was 5 μL with a flow rate of 1.0 mL/min.

## Results and discussion

### Establishing the resveratrol biosynthesis baseline in R. toruloides

**Fig. 2:**
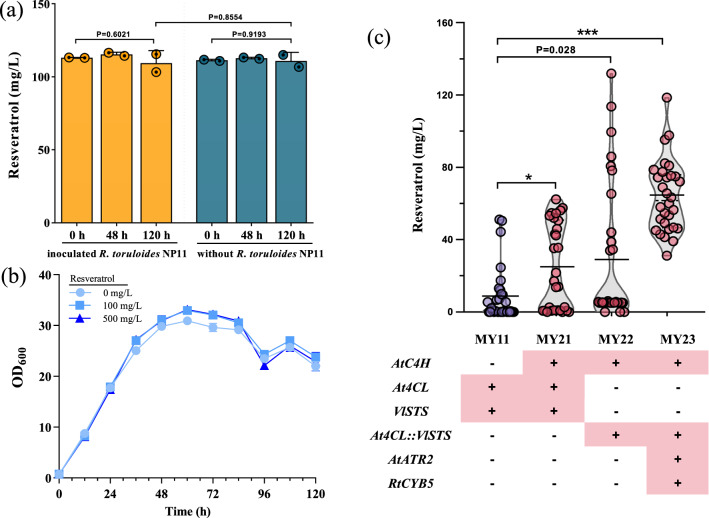
Establishing and optimizing the heterogenous resveratrol biosynthesis pathway. **a** Degradation assays for resveratrol in strain *R. toruloides* NP11. The 0.5 mM (114.13 mg/L) resveratrol was added as a single carbon source. The inoculated group was shown in yellow, and the control group was shown in blue. **b** Toxicity tolerance test for resveratrol in strain *R. toruloides* NP11. The 100 and 500 mg/L resveratrol was added to the YPD medium. **c** is the resveratrol production in engineered strains after 96 h cultivation in YPD medium. The population performance of each engineered strain was quantified using a violin plot. The resveratrol titer of each transformant was shown as circles, and the grey outline represented the density. The black line presents the mean value of each transformant. Statistical significance was analyzed using two-tailed unpaired t-test (*p < 0.05, **p < 0.01, ***p < 0.001)

*R. toruloides* is endowed with a versatile metabolism capability and a wide feedstock spectrum, especially it can efficiently assimilate the resveratrol precursor *p*-CA [10, 17]. To investigate the feasibility of recruiting the bifunctional *Rt*PAL for biosynthesizing resveratrol, the stability of resveratrol was tested during fermentation with *R. toruloides*. Thus, resveratrol was used as the sole carbon source to replace the glucose in YPD (Fig. 2). The resveratrol presented no obvious decrease during the 120 h fermentation, demonstrating that *R. toruloides* would not degrade resveratrol (Fig. 2a). Then, the influence of resveratrol on the growth of *R. toruloides* was investigated. The cell growth was not affected when 500 mg/L resveratrol was added (Fig. 2b). The above results indicated it is possible to harness the *Rt*PAL for biosynthesizing resveratrol in *R. toruloides*.

The *At*4CL and grape derived STS have been extensively utilized for the heterologous production of resveratrol [12]. Here, the two essential enzymes *At*4CL and *Vl*STS were introduced into *R. toruloides*, which were mediated by a P2A peptide, by putting them under the promoter of *pXYL*. The resulting strains (MY11) harboring *At*4CL and *Vl*STS produced resveratrol with an averaged titer of 8.7 mg/L at 96 h (Fig. 2c). Since resveratrol can also be produced from L-Phe by *Rt*PAL in *R. toruloides*, a truncated *A. thaliana* C4H (the N-terminal membrane anchor region, 1–22 amino acid residue, was removed to generate *Att*C4H) was subsequently introduced, which was proved beneficial in supporting resveratrol biosynthesis with other microbial hosts [18] (Fig. 2c). By simultaneously introducing of *Att*C4H, *At*4CL and *Vl*STS, the resultant average resveratrol titer was increased by 176% in the strain group MY21 (24.1 mg/L) (Fig. 2c). The results implied that a synergy between the L-Tyr and L-Phe dependent routes might exist as reported in *S. cerevisiae,* where the L-Phe and L-Tyr routes were combined for producing the aromatic chemicals [19]. The results here also indicated that L-Phe based resveratrol biosynthesis route was more efficient than the one on L-Tyr in *R. toruloides*.

### Enhancing resveratrol production via fusing protein and improving P450 activity

The resveratrol biosynthesis pathway involves two requisite but unstable intermediates, *p*-CA and *p*-coumaroyl-CoA. Protein fusion is a common strategy to facilitate substrate trafficking, avoid metabolic flux leakage, and improve enzymatic efficiency [20, 21]. It has been reported successful in improving the efficiency of substrate delivery to support the resveratrol production by employing the fusion protein 4CL::STS [22, 23]. Therefore, the *At*C4H and the fusion protein *At*4CL::*Vl*STS (linked by Gly-Ser-Gly) were introduced into *R. toruloides* NP11. The resulting strain group MY22 obtained 29.0 mg/L of resveratrol on average, a 20% increase compared with their independent expression in strain group MY21 (Fig. 2c).

The heterologous expression of a plant originated pathway may function sub-optimally due to the unsuitable cofactor as in the case of microbial production of resveratrol [5]. Particularly, the *At*C4H employed in resveratrol synthesis pathway is a membrane-associated plant-derived P450 enzyme, whose heterologous expression may suffer from insufficient cofactor NADPH supply [24]. Additionally, as a heme-thiolate protein, the plant-derived cytochrome P450 monooxygenase *At*C4H also requires a cytochrome P450 redox partner [24, 25]. It has been reported that the decline in the catalytic activity of P450 is caused by inadequate and inefficient cofactors, and it would lead to a limitation of resveratrol overproduction [7, 19]. Accordingly, *At*C4H may need to be remedied in low activity by increasing the electron transfer efficiency. Thus, the P450-mediated redox partner *At*ATR2 was introduced and the endogenous heme prosthetic group *Rt*CYB5 was overexpressed in strain MY22-No.29 (one of the most efficient producer in group MY22) to generate strain group MY23, the average resveratrol production of 30 transformants in the resulting strain group MY23 was improved to 64.1 mg/L, a 121% increase compared to those of MY22 (Fig. 2c). As anticipated, accelerating the catalytic cycle in P450 can effectively increase resveratrol production. The result here was consistent with the previous report where the production of resveratrol by *S. cerevisiae* also increased by about 150% via enhancing the P450 activity [7].

### Validating the critical steps in the shikimic acid and AAAs pathways

**Fig. 3:**
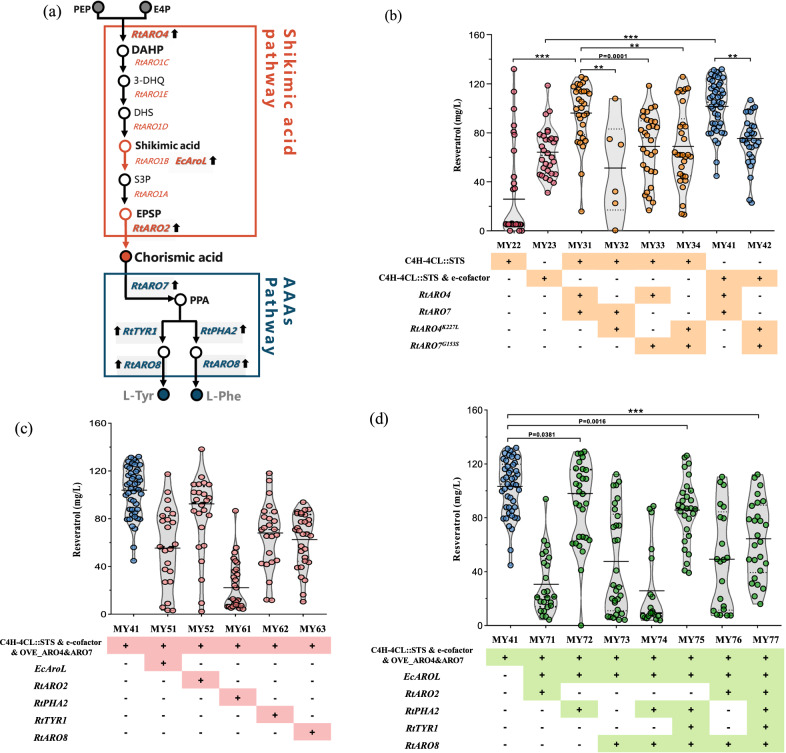
Validating the critical steps within the shikimic acid and AAAs pathways for resveratrol production. **a** Schematic illustration of metabolites and the shikimic acid and AAAs pathways in *R. toruloides*. **b**, **c** and **d** demonstrates resveratrol production in engineered strains after 96 h cultivation in YPD medium by enhancing the shikimic acid pathway **b** and AAAs pathway **c** and their combinations **d** in YPD medium. 3-DHQ, 3-dehydroquinic acid; DHS, 3-dehydroshikimic acid; S3P, shikimic acid 3-phosphate; EPSP, 5-enolpyruvylshikimate 3-phosphate; *Rt*ARO1, pentafunctional AROM polypeptide from *R. toruloides*; *Ec*AroL, shikimate kinase from *E. coli*; *Rt*Aro2, chorismate synthase from *R. toruloides*; *Rt*TYR1, prephenate dehydrogenase from *R. toruloides*; *Rt*PHA2, prephenate dehydrogenase from *R. toruloides*; *Rt*ARO8, aromatic amino acid aminotransferase I from *R. toruloides*

Due to the multibranch and multistep metabolic pathway, it is challenging for microbial overproduction of plant secondary metabolites [26]. In this case, the critical enzymes like shikimate kinase, chorismate synthase *ARO2*, prephenate dehydratase *PHA2*, prephenate dehydrogenase *TYR1*, and aromatic amino acid aminotransferase I *ARO8* in the shikimate acid and AAAs pathways have been reported as the potential limiting steps for further boosting resveratrol production (Fig. 3a) [18].

A sophisticated and strict metabolic network regulates the biosynthesis pathway of aromatics, especially the feedback inhibition of aromatic amino acids on ARO4 (the first enzyme of the shikimic acid pathway) and ARO7 (the route point enzyme of the AAAs pathway) [11, 27, 28]. First, the potential mutation sites of Aro4p and Aro7p in *R. toruloides* were identified by multiple pairwise sequence alignment to their counterpart in *S. cerevisiae* and *Y. lipolytica*. Then, the single-point mutations were introduced into the wild-type proteins to obtain the feedback-insensitive mutant enzymes *Rt*ARO4^K227L^ and *Rt*ARO7^G153S^. Subsequently, plasmids harboring combinations of the wild-type *Rt*ARO4 and *Rt*ARO7 and the feedback-insensitive mutants *Rt*ARO4^K227L^ and *Rt*ARO7^G153S^ were constructed (Fig. 3b).

Next, the above four recombinant plasmids were introduced into the strain MY22-No.29, resulting in the engineered strain groups of MY31, MY32, MY33, and MY34 (Fig. 3b), which have shown a sharp increase in the average production of resveratrol by 233% (96.5 mg/L), 137% (68.7 mg/L), 138% (68.9 mg/L) and 78% (51.4 mg/L) in comparison with an average production of the parental strain group MY22 respectively. Interestingly, the production capacity of strain group MY31, which carried the wild-type *Rt*ARO4 and *Rt*ARO7, was significantly higher (p < 0.05) than that of strain group MY34, which bearing the feedback-insensitive mutants *Rt*ARO4^K227L^ and *Rt*ARO7^G153S^. The wild-type *Rt*ARO4 and *Rt*ARO7 were also overexpressed in the strain MY23-No.26 (the highest yield transformant in group MY23) to form the resulting strain group MY41, which produced an average of 102.6 mg/L resveratrol. Likewise, the mutants *Rt*ARO4^K227L^ and *Rt*ARO7^G153S^ were also introduced into strain MY23-No.26 to obtain strain group MY42, whose averaged resveratrol titer reached 76.1 mg/L (Fig. 3b). The results showed that relieving feedback inhibition regulation could increase resveratrol production while raising the expression of *Rt*ARO4 and *Rt*ARO7 showed a more positive effect on resveratrol overproduction (125.2 mg/L).

Although it may seem counterintuitive, this inconsistency may be due to the reasons listed below. (1) The low accumulation of 3-deoxy-arabino-heptulonate-7-phosphate (DAHP) and aromatic amino acids was insufficient to initiate concentration-dependent negative feedback inhibition. (2) The catalytic activity of *Rt*ARO4^K227L^ and *Rt*ARO7^G153S^ cannot surpass that of the wild types after introducing the point mutation at the regulatory site. (3) Due to the limitations of the genetic manipulation technique, the interference caused by the background expression of endogenous *Rt*ARO4 and *Rt*ARO7 could not be avoided.

Furthermore, previous researches indicated that the shikimate kinase AroL and the chorismate synthase ARO2 might be restricted factors in the shikimate pathway [29, 30]. Thus, the heterologous *Ec*AroL from *E. coli* and the endogenous *Rt*ARO2 were overexpressed in MY41-No.41, respectively. However, the resveratrol production in the resulting strain groups MY51 and MY52 were decreased (Fig. 3c). Likewise, the overexpression of the potential critical enzymes, including prephenate dehydratase *Rt*PHA2, prephenate dehydrogenase *Rt*TYR1, and aromatic amino acid aminotransferase I *Rt*ARO8, also resulted in decreased production of resveratrol (Fig. 3c). Unexpectedly, overexpression of seven combinations of the above five genes showed a significant adverse effect on resveratrol production (p < 0.05) (Fig. 3d).

Clearly, the results here were quite beyond anticipation, for which the possible explanations are as follows: (1) There might be a remained unclear and harsh regulatory system in *R. toruloides* which inhibited the positive effect on resveratrol production by single-mindedly increasing expression levels; for example, the regulation mechanism of the enzyme catalytic activity based on substrate concentration [19, 30]. (2) The current metabolic bottlenecks may be elsewhere, for example, low metabolic flux from the central metabolism into the shikimic acid pathway.

### The effects of cerulenin on resveratrol production

**Fig. 4:**
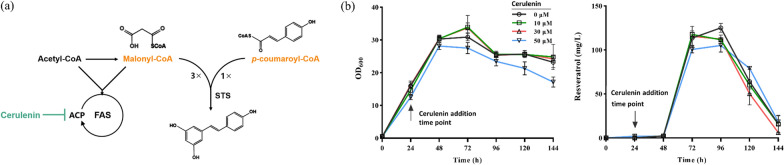
The effects of cerulenin on resveratrol production in *R. toruloides*. **a** Schematic illustration of the cerulenin effects on resveratrol production. **b** The OD_600_ and resveratrol production of strain MY41-No.41 under different concentrations of cerulenin. All data indicated the mean of n = 3 biologically independent samples, and error bars show standard deviation. **c** Time profile of resveratrol production, pH, glucose and OD_600_ from strain MY41-No.41 under different conditions. The red lines denote additions of 0.1 mM citrate buffer (pH = 6.0), and the blue lines denote the control group

Generally, malonyl-CoA is considered the rate-limiting step in resveratrol synthesis since each molecule of resveratrol consumes three molecules of malonyl-CoA [31, 32]. As an oleaginous yeast, there might be more competition for malonyl-CoA between the biosynthesis of resveratrol and lipids [33]. Therefore, cerulenin, an efficient FAS inhibitor, was added to determine whether malonyl-CoA is the bottleneck in resveratrol biosynthesis at the current stage [34] (Fig. 4a).

The highest resveratrol producing strain MY41-No.41 was utilized by supplementing different concentrations of cerulenin (0 μM, 10 μM, 30 μM, 50 μM) into the cultivation medium after 24 h incubation (OD_600_ = 15–20). As shown in Fig. 4b, strain MY41-No.41 produced 125.2 mg/L resveratrol without the addition of cerulenin, which was significantly higher than those obtained with the addition of cerulenin (111.6 mg/L with 10 μM (p = 0.0119), 112.4 mg/L with 30 μM (p = 0.0324) and 105.0 mg/L with 50 μM (p = 0.0175)). This decline in resveratrol production might be due to the disturbed cell state aroused by lipid metabolism [31]. Moreover, there was an observable growth inhibition when 50 μM cerulenin was added, which may be caused by the fact that lipid metabolism is necessary for cell growth [31]. The results indicated that malonyl-CoA might be adequate in engineered strain for supporting resveratrol synthesis.

## Conclusions

This is the first report on engineering *R. toruloides* for resveratrol production, which was achieved by recruiting heterologous *At*C4H, *At*4CL, and *Vl*STS. The resveratrol production was enhanced via protein fusion, cofactor manipulation, and *ARO4* and *ARO7* overexpression. The best producer MY41-No.41 produced 125.2 mg/L in the 250 mL flask from the YPD medium. The present work would provide a reference for the further exploration of *R. toruloides* as a platform for phenylpropanoids production.

## Supplementary Information

**Additional file 1: Table S1.** Strain and plasmids used in this work. **Table S2.** Main primers used in this work. **Table S3.** The highest resveratrol yield among the different hosts and their engineering strategies. **Figure S1.** Validation of resveratrol production in engineered strain by LCMS. The characteristic peaks of the resveratrol standard are at m/z=227.0714 and m/z=228.0751, and that of fermentation extracts from engineered strain is at m/z=227.0716 and m/z=228.0749, which is highly consistent within the standard. **Figure S2.** Multiple pairwise sequence alignment between *S. cerevisiae*, *Y. lipolytica* and *R. toruloides* Aro4p and Aro7p. Multiple pairwise alignments of Aro4p; In *Sc*Aro4p, the 229th amino acid resulted in a feedback insensitivity when lysine mutated into leucine (Hartmann et al., 2003), and the 211th amino acid in *Yl*Aro4p has the same mutation (Palmer et al., 2020), which are marked with red arrows. For Aro7p, In *Sc*Aro7p, amino acid 141 resulted in a non-allosterically regulated when glycine mutated into serine (Schnappauf et al., 1998), and the 139th amino acid in *Yl*Aro7p has the same mutation (Sáez-Sáez et al., 2020) which are marked with red arrows. **Figure S3.** Fluorescence microscopy analysis of lipids in engineered *R. toruloides* stained with Nile Red. Scale bars, 20 μM.

## Data Availability

E-supplementary data for this work can be found in the e-version of this paper online. The datasets used and/or analyzed during the current study are available from the corresponding author on reasonable request.
