# Supplementary material for: Metabolic engineering of Rhodotorula toruloides for resveratrol production
Source: Microb Cell Fact. 2022 Dec 24;21:270. doi: 10.1186/s12934-022-02006-w (PMC9789595; doi:10.1186/s12934-022-02006-w)
Supplement: Supplementary file 1 — Additional file 1: Table S1. Strain and plasmids used in this work. Table S2. Main primers used in this work. Table S3. The highest resveratrol yield among the different hosts and their engineering strategies. Figure S1. Validation of resveratrol production in engineered strain by LCMS. The characteristic peaks of the resveratrol standard are at m/z=227.0714 and m/z=228.0751, and that of fermentation extracts from engineered strain is at m/z=227.0716 and m/z=228.0749, which is highly consistent within the standard. Figure S2. Multiple pairwise sequence alignment between S. cerevisiae, Y. lipolytica and R. toruloides Aro4p and Aro7p. Multiple pairwise alignments of Aro4p; In ScAro4p, the 229th amino acid resulted in a feedback insensitivity when lysine mutated into leucine (Hartmann et al., 2003), and the 211th amino acid in YlAro4p has the same mutation (Palmer et al., 2020), which are marked with red arrows. For Aro7p, In ScAro7p, amino acid 141 resulted in a non-allosterically regulated when glycine mutated into serine (Schnappauf et al., 1998), and the 139th amino acid in YlAro7p has the same mutation (Sáez-Sáez et al., 2020) which are marked with red arrows. Figure S3. Fluorescence microscopy analysis of lipids in engineered R. toruloides stained with Nile Red. Scale bars, 20 μM. [file 12934_2022_2006_MOESM1_ESM.docx]

**Additional file Information**

**Metabolic engineering of *Rhodotorula toruloides* for resveratrol production**

Mengyao Zhang^1^, Xiaobing Yang^1,^*

* Corresponding authors

Xiaobing Yang

College of Enology, Northwest A&F University, Yangling, Shaanxi 712100, China

E-mail: yangxb@nwafu.edu.cn

**Table S1. Strain and plasmids used in this work**

| Strain/Plasmid | Relevant genotype | Origin |
| --- | --- | --- |
| Strain | | |
| *E.coli* DH5α | For plasmid clone | Takara |
| *A.tumefaciens* AGL1 | For transformation | (Lin et al., 2014) |
| *R. toruloides* NP11 | Wild type, MAT A1 | (Zhu et al., 2012) |
| MY11 | MAT A1, *At4CL*-*VlSTS*, Ntc^R^ | This work |
| MY21 | MAT A1, *AtC4H*-*At4CL*-*VlSTS*, Ntc^R^ | This work |
| MY22 | MAT A1, *AtC4H*-*At4CL*::*VlSTS*, Ntc^R^ | This work |
| MY23 | MAT A1, *AtC4H*-*At4CL*::*VlSTS* -*AtATR2*-*RtCYB5*, Ntc^R^, Hyg^R^ | This work |
| MY31 | *MAT A1, AtC4H-At4CL::VlSTS-RtARO4-RtARO7*, Ntc^R^, Hyg^R^ | This work |
| MY32 | *MAT A1, AtC4H-At4CL::VlSTS-RtARO4^K227L^-RtARO7*, Ntc^R^, Hyg^R^ | This work |
| MY33 | *MAT A1, AtC4H-At4CL::VlSTS-RtARO4-RtARO7^G153S^*, Ntc^R^, Hyg^R^ | This work |
| MY34 | *MAT A1, AtC4H-At4CL::VlSTS-RtARO4^K227L^-RtARO7^G153S^*, Ntc^R^, Hyg^R^ | This work |
| MY41 | *MAT A1, AtC4H-At4CL::VlSTS-RtARO4-RtARO7-AtATR2-RtCYB5*, Ntc^R^, Hyg^R^ | This work |
| MY42 | *MAT A1, AtC4H-At4CL::VlSTS-RtARO4^K227L^-RtARO7^G153S^*-*AtATR2-RtCYB5*, Ntc^R^, Hyg^R^ | This work |
| MY51 | *MAT A1, AtC4H-At4CL::VlSTS-RtARO4-RtARO7-AtATR2-RtCYB5-RtARO2*, Ntc^R^, Hyg^R^, Ble^R^ | This work |
| MY52 | *MAT A1, AtC4H-At4CL::VlSTS-RtARO4-RtARO7-AtATR2-RtCYB5-RtPHA2*, Ntc^R^, Hyg^R^, Ble^R^ | This work |
| MY61 | *MAT A1, AtC4H-At4CL::VlSTS-RtARO4-RtARO7-AtATR2-RtCYB5-RtTYR1*, Ntc^R^, Hyg^R^, Ble^R^ | This work |
| MY62 | *MAT A1, AtC4H-At4CL::VlSTS-RtARO4-RtARO7-AtATR2-RtCYB5-RtARO8*, Ntc^R^, Hyg^R^, Ble^R^ | This work |
| MY63 | *MAT A1, AtC4H-At4CL::VlSTS-RtARO4-RtARO7-AtATR2-RtCYB5-EcAROL*, Ntc^R^, Hyg^R^, Ble^R^ | This work |
| MY71 | *MAT A1, AtC4H-At4CL::VlSTS-RtARO4-RtARO7-AtATR2-RtCYB5-EcAROL-RtARO2*, Ntc^R^, Hyg^R^, Ble^R^ | This work |
| MY72 | *MAT A1, AtC4H-At4CL::VlSTS-RtARO4-RtARO7-AtATR2-RtCYB5-EcAROL-RtPHA2*, Ntc^R^, Hyg^R^, Ble^R^ | This work |
| MY73 | *MAT A1, AtC4H-At4CL::VlSTS-RtARO4-RtARO7-AtATR2-RtCYB5-EcAROL-RtARO8*, Ntc^R^, Hyg^R^, Ble^R^ | This work |
| MY74 | *MAT A1, AtC4H-At4CL::VlSTS-RtARO4-RtARO7-AtATR2-RtCYB5-EcAROL-RtPHA2-RtARO8*, Ntc^R^, Hyg^R^, Ble^R^ | This work |

(continue Table S1)

| Strain/Plasmid | Relevant genotype | Origin |
| --- | --- | --- |
|  |  |  |
| MY75 | *MAT A1, AtC4H-At4CL::VlSTS-RtARO4-RtARO7-AtATR2-RtCYB5-EcAROL-RtPHA2-RtTYR1-RtARO8*, Ntc^R^, Hyg^R^, Ble^R^ | This work |
| MY76 | *MAT A1, AtC4H-At4CL::VlSTS-RtARO4-RtARO7-AtATR2-RtCYB5-EcAROL-RtARO2-RtARO8*, Ntc^R^, Hyg^R^, Ble^R^ | This work |
| MY77 | *MAT A1, AtC4H-At4CL::VlSTS-RtARO4-RtARO7-AtATR2-RtCYB5-EcAROL-RtARO2-RtPHA2-RtTYR1-RtARO8*, Ntc^R^, Hyg^R^, Ble^R^ | This work |
| Plasmid | | |
| pUC57 | For gene amplification, Kan^R^ | Synbio |
| pET-28a(+) | For gene amplification, Amp^R^ | Synbio |
| pZPK | Template plasmid, binary vector, Kan^R^ | (Lin et al., 2014) |
| pZPK-LS | pZPK, Ppgk-Ntc^R^-Tnos, pXYL*-At4CL-VlSTS-*Thsp | This work |
| pZPK-HLS | pZPK, Ppgk-Ntc^R^-Tnos, pXYL*-AtC4H-At4CL-VlSTS-*Thsp | This work |
| pZPK-HL::S | pZPK, Ppgk-Ntc^R^-Tnos, pXYL*-AtC4H-At4CL::VlSTS-*Thsp | This work |
| pZPK-AC | pZPK, Ppgk-Hyg^R^-Tnos, pXYL*-AtATR2-RtCYB5-*Thsp | This work |
| pZPK-AR47NM | pZPK, Ppgk-Hyg^R^-Tnos, Pxyl-*RtARO4-RtARO7-*Thsp | This work |
| pZPK-AR47IM | pZPK, Ppgk-Hyg^R^-Tnos, Pxyl-*RtARO4^K227L^-RtARO7-*Thsp | This work |
| pZPK-AR47SM | pZPK, Ppgk-Hyg^R^-Tnos, Pxyl-*RtARO4-RtARO7^G153S^-*Thsp | This work |
| pZPK-AR47DM | pZPK, Ppgk-Hyg^R^-Tnos, Pxyl-*RtARO4^K227L^-RtARO7^G153S^-*Thsp | This work |
| pZPK-AR47DMAC | pZPK, Ppgk-Hyg^R^-Tnos, Pxyl-*RtARO4^K227L^-RtARO7^G153S^-AtATR2-RtCYB5-*Thsp | This work |
| pZPK-AR47NMAC | pZPK, Ppgk-Hyg^R^-Tnos, Pxyl-*RtARO4-RtARO7-AtATR2-RtCYB5-*Thsp | This work |
| pZPK-AROL | pZPK, Ppgk-Ble^R^-Tnos, Pxyl-*EcAroL-*Thsp | This work |
| pZPK-ARO2 | pZPK, Ppgk-Ble^R^-Tnos, Pxyl-*RtARO2-*Thsp | This work |
| pZPK-PHA2 | pZPK, Ppgk-Ble^R^-Tnos, Pxyl-*RtPHA2-*Thsp | This work |
| pZPK-TYR1 | pZPK, Ppgk-Ble^R^-Tnos, Pxyl-*RtTYR1-*Thsp | This work |
| pZPK-ARO8 | pZPK, Ppgk-Ble^R^-Tnos, Pxyl-*RtARO8-*Thsp | This work |
| pZPK-L2 | pZPK, Ppgk-Ble^R^-Tnos, Pxyl-*EcAroL-RtARO2-*Thsp | This work |
| pZPK-LP | pZPK, Ppgk-Ble^R^-Tnos, Pxyl-*EcAroL-RtPHA2-*Thsp | This work |
| pZPK-8L | pZPK, Ppgk-Ble^R^-Tnos, Pxyl*-RtARO8*-*EcAroL -*Thsp | This work |
| pZPK-8LP | pZPK, Ppgk-Ble^R^-Tnos, Pxyl-*RtARO8-EcAroL-RtPHA2*-Thsp | This work |
| pZPK-8LPT | pZPK, Ppgk-Ble^R^-Tnos, Pxyl*-RtARO8-EcAroL-RtPHA2- RtTYR1*-Thsp | This work |
| pZPK-CAE | pZPK, Ppgk-Ble^R^-Tnos, Pxyl*-RtARO8*-*EcAroL-RtARO2-*Thsp | This work |
| pZPK-CAEPT | pZPK, Ppgk-Ble^R^-Tnos, Pxyl*-RtARO8*-*EcAroL-RtARO2-RtPHA2-RtTYR1*-Thsp | This work |

**Table S2. Main primers used in this work**

| Primer | | Sequence (5’-3’) | | | |
| --- | --- | --- | --- | --- | --- |
| For amplification | | | | | |
| 4CL-EcoRV-F | | | | CCGATATCATGGCTCCCCAGGAGCAGGCTG | |
| STS-SpeI-R | | | | GGACTAGTCTAGTGGTGGTGGTGGTGGTGG | |
| C4H-EcoRV-F | | | | CCGATATCATGTCGAAGCTCCGCGGCAAG | |
| ARO7-EcoRV-F | | | | CCGATATCATGAACTTCACCGCAGGACG | |
| ARO4-SpeI-R | | | | GGACTAGTCTAAGCAGCCGGGAACTGCGC | |
| ARO7-fu-XYL-F | | | | CGCAGAATACACGCCGATATCATGAACTTCACCGCAGG | |
| ATR2-EcoRV-F | | | | CCGATATCCGCCGCTCTGGCTCGGGCAACTCG | |
| ATR2-fu-ARO4-F | | | | CAGTTCCCGGCTGCTGGCTCGGGAGCGACCAACTTCTCGC | |
| ATR2-fu-CYB5-R | | | | GGCCCTCGCCGGAACCCCAGACGTCGCGGAGGTAGCGACC | |
| CYB5-fu-ATR2-R | | | | CCTCCGCGACGTCTGGGGTTCCGGCGAGGGCCGCGGCTC | |
| CYB5-SpeI-R | | | | GGACTAGTCTACTGCGAGACGCGCCCGACGATG | |
| CYB5-fu-tHSP-R | | | | GACGGGGCGGAATCGTACTAGTCTACTGCGAGACGCGCC | |
| For verification |  | | | |  |
| tHSP-R | | | | CGGAAGAGAGGAAAAGCGGACGACTG |  |
| XYL-F | | | | GCAGGTGCGTGGCATCGTATA |  |
| 47AC-3872bp-F | | | | CCTCTTCTTCGGCTGCCGCAACC |  |
| ATR2-799bp-F | | | | CAACATGGCGAACGGCAACGGCTACAC |  |
| ARO4-1907bp-F | | | | CAGAGTATTCCAGCGAACGGACCGC |  |
| HL-fu-STS-3051bp-F | | | | GCTCTCGGAGGACGACGTCAAGCAG |  |
| For chimeric enzyme | | | | |  |
| C4H-fu-XYL-F | | | | CACGCAGAATACACGCCGATATCATGTCGAAGCTCCGCGGC |  |
| 4CL-fu-STS-R | | | | GACGGGGCGGAATCGTACTAGTCTAGCCCGAGCCGAGGCCGTTGGCGAGCTTC |  |
| STS-fu-4CL-F | | | | GGCCTCGGCTCGGGCGGCTTCGGTCGAGGAGTTCCGC |  |
| STS-fu-thsp-R | | | | GACGGGGCGGAATCGTACTAGTCTAGTGGTGGTGGTGGTGGTG |  |
| For mutation | | |  | |  |
| ARO7-G153S-F | | | | CCGATATCGGAATTATTCGAGTGCCGGGAC |  |
| ARO7-fu-insert-R | | | | CACTCGAATAATTCCCATCGTCGTTCTCCTTGCCCAGC |  |
| ARO4-L227K-EcoRV-F | | | | CCGATATCTCCGTCACCAAGCAGGGAATCTC |  |
| ARO4-fu-insert2-R | | | | CTGGAGGGTGACGGAGAGGAACGAGTGAGAGGCGGCC |  |

**Table S3. The** **highest resveratrol yield among the different hosts and their engineering strategies**.

| Host strain | Engineering strategy | Substrate | Cultivation mode | Titer (mg/L) | Productivity (mg/L/h)^a^ | Yield (mg/g) | Reference |
| --- | --- | --- | --- | --- | --- | --- | --- |
| *Escherichia coli* BW27784 | - Expressed genes encoding *At*4CL and *Vv*STS | p-Coumaric acid and cerulenin | Shake flask | 2340 | 97.5 | NA | [5] |
| *C. glutamicum DelAro3* | - Expressed genes encoding *Pc*4CL and *Ah*STS - Deletion of phdB, pcaF and pobA | p-coumaric acid and cerulenin | Shake flask | 158 | NA | NA | [2] |
| *S. venezuelae* DHS2001 | - Expressed genes encoding *Sc*4CL and *Ah*STS - Deletion of pks | *p*-coumaric acid | Shake flask | 0.4 | NA | NA | [11] |
| *Synechococcus elongatus* PCC7942 | - Expressed genes encoding aroG^fbr^, sam8 and 4CL::STS | CO_2_ and cerulenin | 100 μmol photons/m^2^/s | 4.6 | 0.02 | NA | [8] |
| *Saccharomyces cerevisiae* CEN. PK102-5B | - Expressed genes encoding AtPAL, *At*C4H, *At*4CL2 and *Vv*VST1 - Overexpression of *Sc*ARO4^K229L^, *Sc*ARO7^G141S^, ACC1, *Sc*CYB5, *At*ATR2 and *Se*ACS - Deletion of ARO10 - Multi-copy integration | Glucose | Fed batch fermentation | 812 | 7.4^b^ | 9.23^a^ | [4] |

**Table S3. (continued)**

| *Yarrowia lipolytica* Po1f | - Expressed genes encoding *Fj*TAL, *Pc*4CL::*Vv*STS, *Sm*PAL, *Sm*C4H, *At*ATR2, *Ca*FPK, *Bs*PTA - Overexpression of *Yl*ARO4^K221L^_,_ *Yl*ARO7^G139S^, *Yl*ARO1, *Yl*CYB5, *Yl*ARO3^K225L^ - Knockout DGA1 - Multi-copy integration | Glucose | 5-L Fed batch fermentation | 22500 | 160.7 | 65.5 | [7] |
| --- | --- | --- | --- | --- | --- | --- | --- |
| *Ogataea polymorpha* | - Expressed genes encoding *Ha*TAL, *At*4CL and *Vv*STS - Multi-copy integration | Tyrosine | Shake flask | 97.2 | NA | NA | [12] |
| *Scheffersomyces stipiti* | - Expressed genes encoding *Ha*TAL, *At*4CL2, *Vv*VST1, *Ss*ARO4^K220L^ and *Ss*ARO7^G139S^ | Sucrose | Shake flask | 668.6 | 5.6^a^ | 13.4^b^ | [3] |
| *Rhodosporidium toruloides* np11 | - Expressed genes encoding *At*C4H and *At*4CL::*Vl*STS - Overexpression of *At*ATR2,   *Rt*CYB5, *Rt*ARO4 and *Rt*ARO7 | Glucose | Shake flask | 125.2 | 1.4 | 6.3 | This work |

NA, not available.

a Calculated by the data presented in the references.

b Calculated by the data estimated from the figure or graph.


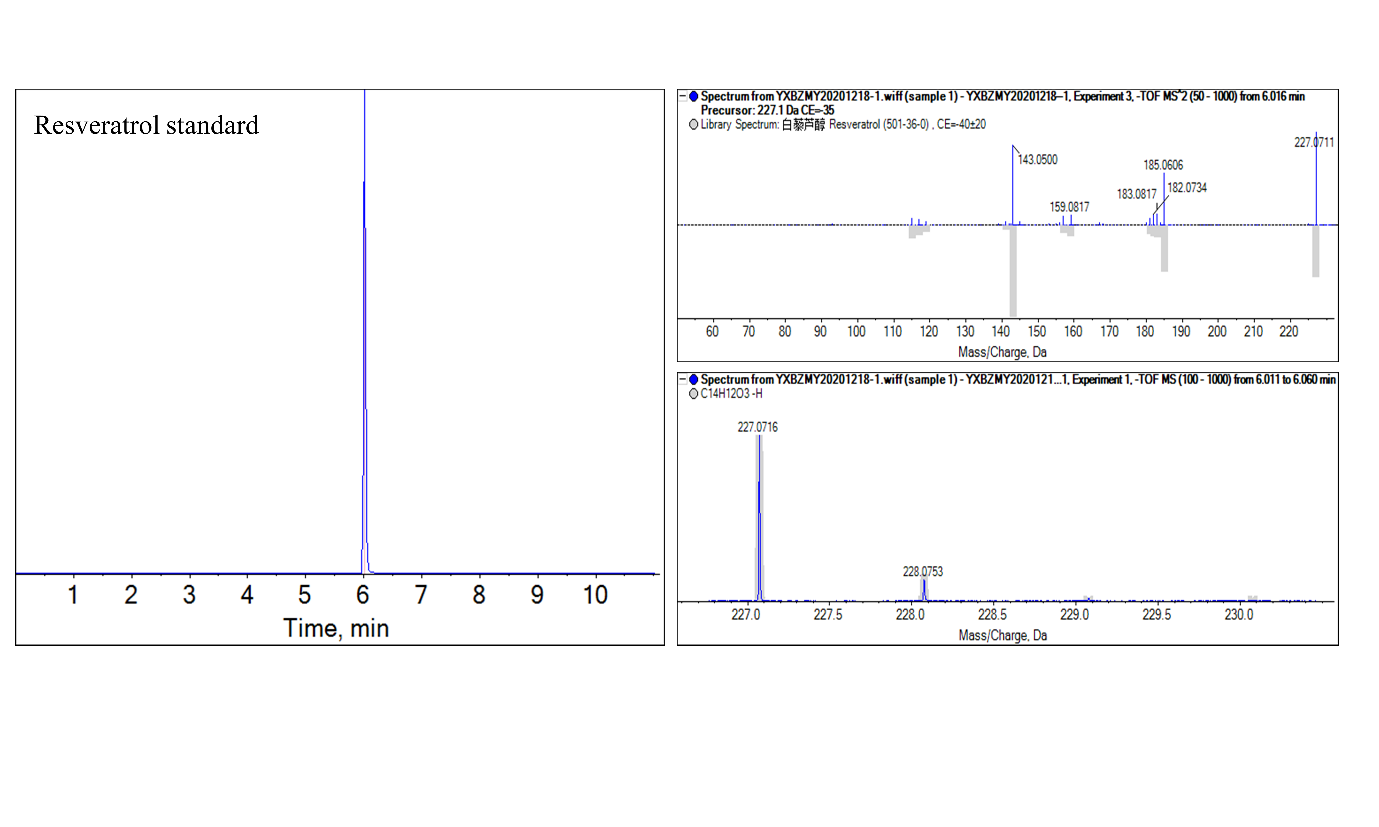

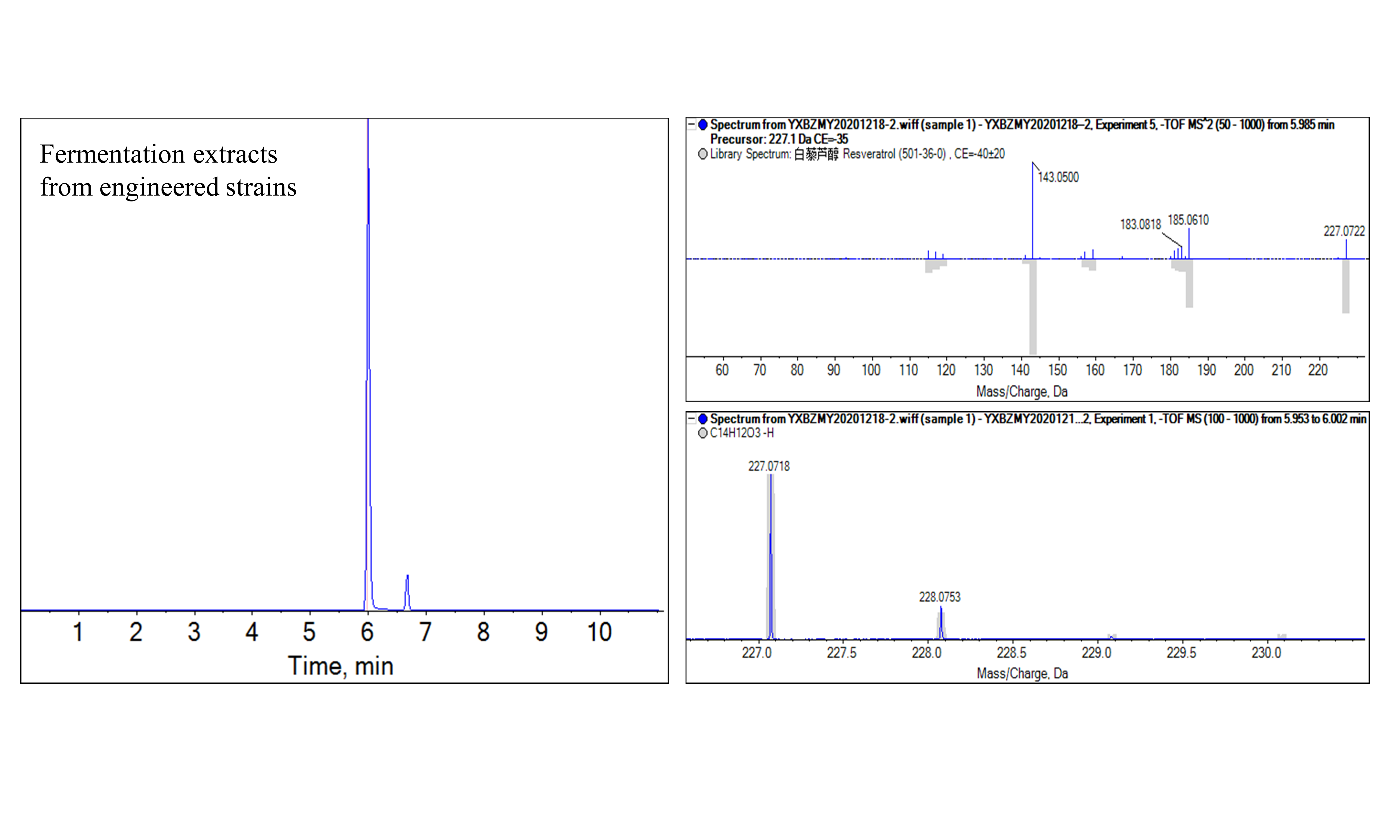


**Fig. S1. Validation of resveratrol production in engineered strain by LCMS.** The characteristic peaks of the resveratrol standard are at m/z=227.0714 and m/z=228.0751, and that of fermentation extracts from engineered strain is at m/z=227.0716 and m/z=228.0749, which is highly consistent within the standard.


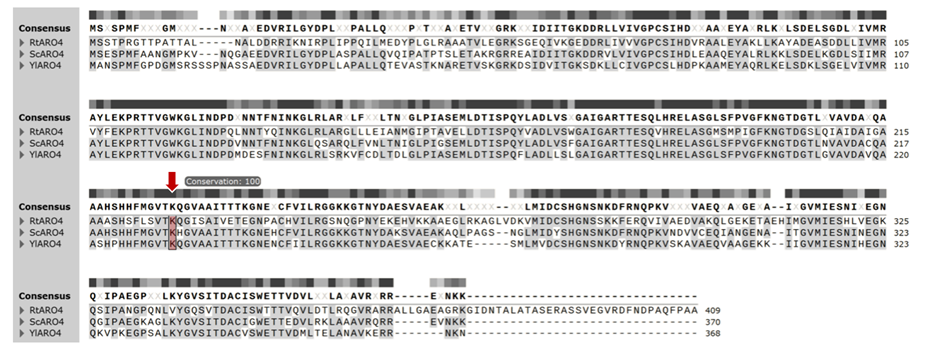


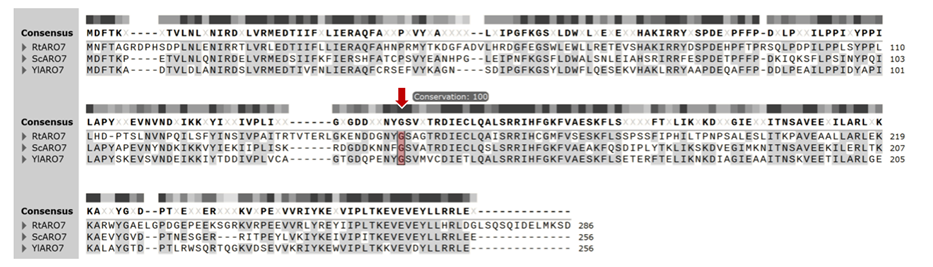


**Fig. S2. Multiple pairwise sequence alignment between *S. cerevisiae*, *Y. lipolytica* and** ***R. toruloides* Aro4p and Aro7p.** Multiple pairwise alignments of Aro4p; In *Sc*Aro4p, the 229th amino acid resulted in a feedback insensitivity when lysine mutated into leucine (Hartmann et al., 2003), and the 211th amino acid in *Yl*Aro4p has the same mutation (Palmer et al., 2020), which are marked with red arrows. For Aro7p, In *Sc*Aro7p, amino acid 141 resulted in a non-allosterically regulated when glycine mutated into serine (Schnappauf et al., 1998), and the 139th amino acid in *Yl*Aro7p has the same mutation (Sáez-Sáez et al., 2020) which are marked with red arrows.


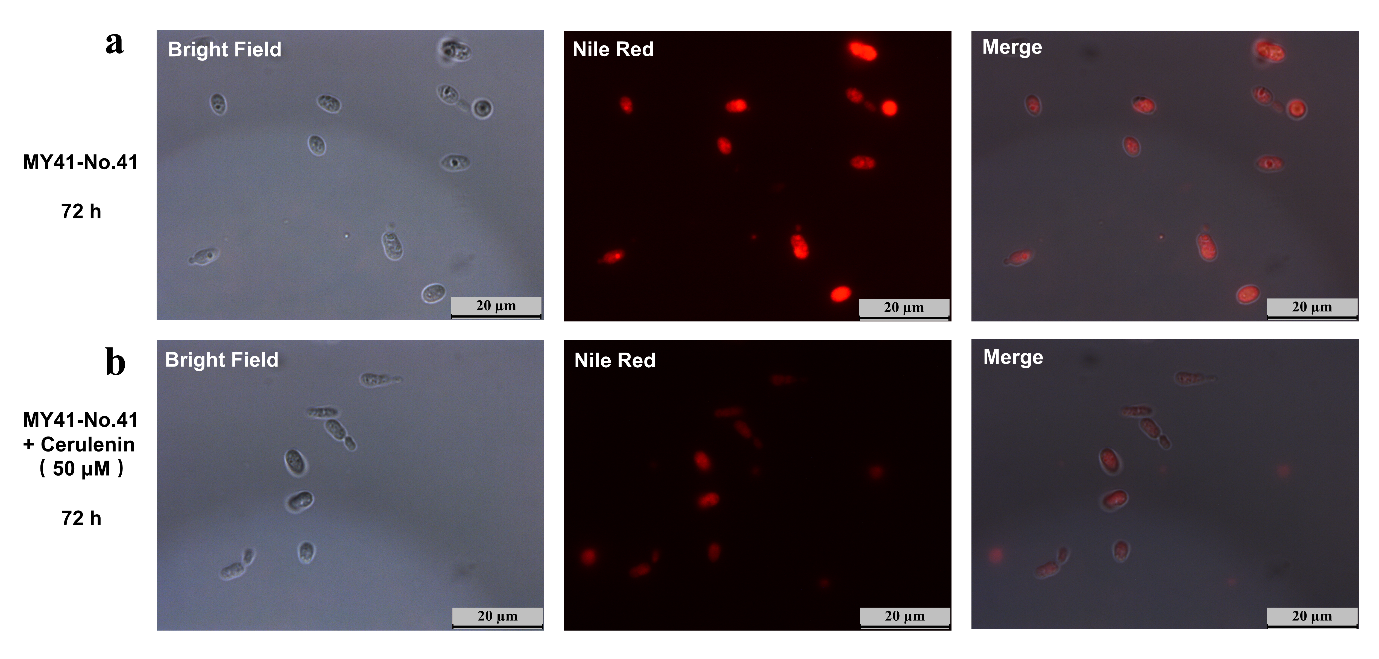


**Fig. S3. Fluorescence microscopy analysis of lipids in engineered *R. toruloides* stained with Nile Red.** Scale bars, 20 μM.

**References**

1. Hartmann, M., Schneider, T.R., Pfeil, A., Heinrich, G., Lipscomb, W.N., Braus, G.H., 2003. Evolution of feedback-inhibited / barrel isoenzymes by gene duplication and a single mutation. Proc. Natl. Acad. Sci. 100, 862–867.
2. Kallscheuer, N., Vogt, M., Stenzel, A., Gätgens, J., Bott, M., Marienhagen, J., 2016. Construction of a *Corynebacterium glutamicum* platform strain for the production of stilbenes and (2S)-flavanones. Metab. Eng. 38, 47–55.
3. Kobayashi, Y., Inokuma, K., Matsuda, M., Kondo, A., Hasunuma, T., 2021. Resveratrol production from several types of saccharide sources by a recombinant *Scheffersomyces stipitis* strain. Metab. Eng. Commun. 13, e00188.
4. Li, M., Schneider, K., Kristensen, M., Borodina, I., Nielsen, J., 2016. Engineering yeast for high-level production of stilbenoid antioxidants. Sci. Rep. 6, 36827.
5. Lim, C.G., Fowler, Z.L., Hueller, T., Schaffer, S., Koffas, M.A.G., 2011. High-yield resveratrol production in Engineered *Escherichia coli*. Appl. Environ. Microbiol. 77, 3451–3460.
6. Lin, X., Wang, Y., Zhang, S., Zhu, Z., Zhou, Y.J., Yang, F., Sun, W., Wang, X., Zhao, Z.K., 2014. Functional integration of multiple genes into the genome of the oleaginous yeast *Rhodosporidium toruloides*. FEMS Yeast Res. 14, 547–555.
7. Liu, M., Wang, C., Ren, X., Gao, S., Yu, S., Zhou, J., 2022. Remodelling metabolism for high-level resveratrol production in *Yarrowia lipolytica*. Bioresour. Technol. 365, 128178.
8. Ni, J., Tao, F., Wang, Y., Yao, F., Xu, P., 2016. A photoautotrophic platform for the sustainable production of valuable plant natural products from CO_2_. Green Chem. 18, 3537–3548.
9. Palmer, C.M., Miller, K.K., Nguyen, A., Alper, H.S., 2020. Engineering 4-coumaroyl-CoA derived polyketide production in *Yarrowia lipolytica* through a *β*-oxidation mediated strategy. Metab. Eng. 57, 174–181.
10. Schnappauf, G., Krappmann, S., Braus, G.H., 1998. Tyrosine and tryptophan act through the same binding site at the dimer interface of yeast chorismate mutase. J. Biol. Chem. 273, 17012–17017.
11. Shang, Y., Huang, S., 2020. Engineering plant cytochrome P450s for enhanced synthesis of natural products: Past achievements and future perspectives. Plant Commun. 1, 100012.
12. Wang, L., Deng, A., Zhang, Y., Liu, S., Liang, Y., Bai, H., Cui, D., Qiu, Q., Shang, X., Yang, Z., He, X., Wen, T., 2018. Efficient CRISPR–Cas9 mediated multiplex genome editing in yeasts. Biotechnol. Biofuels 11, 277.
13. Zhu, Z., Zhang, S., Liu, H., Shen, H., Lin, X., Yang, F., Zhou, Y.J., Jin, G., Ye, M., Zou, H., Zhao, Z.K., 2012. A multi-omic map of the lipid-producing yeast *Rhodosporidium toruloides*. Nat. Commun. 3, 1112.
